# A model to predict nodal metastasis in patients with oral squamous cell carcinoma

**DOI:** 10.1371/journal.pone.0201755

**Published:** 2018-08-09

**Authors:** R. K. De Silva, B. S. M. S. Siriwardena, A. Samaranayaka, W. A. M. U. L. Abeyasinghe, W. M. Tilakaratne

**Affiliations:** 1 Department of Oral Diagnostic and Surgical Sciences, Faculty of Dentistry, University of Otago, Dunedin, New Zealand; 2 Department of Oral Pathology, Faculty of Dental Sciences, University of Peradeniya, Peradeniya, Sri Lanka; 3 Department of Preventive and Social Medicine, Faculty of Medicine, University of Otago, Dunedin, New Zealand; Rajiv Gandhi Centre for Biotechnology, INDIA

## Abstract

Difficulty in precise decision making on necessity of surgery is a major problem when managing oral squamous cell carcinomas (OSCCs) with clinically negative neck. Therefore, use of clinical and histopathological parameters in combination would be important to improve patient management. The main objective is to develop a model that predicts the presence of nodal metastasis in patients with OSCC.623 patients faced neck dissections with buccal mucosal or tongue squamous cell carcinoma (SCC) were selected from patients’ records. Demographic data, clinical information, nodal status, Depth of invasion (DOI) and pattern of invasion (POI) were recorded. The parameters which showed a significant association with nodal metastasis were used to develop a multivariable predictive model (PM). Univariate logistic regression was used to estimate the strengths of those associations in terms of odds ratios (OR). This showed statistically significant associations between status of the nodal metastasis and each of the following 4 histopathological parameters individually: size of the tumour (T), site, POI, and DOI. Specifically, OR of nodal metastasis for tongue cancers relative to buccal mucosal cancers was 1.89, *P*-value < 0.001. Similarly, ORs for POI type 3 and 4 relative to type 2 were 1.99 and 5.83 respectively. A similar relationship was found with tumour size; ORs for T2, T3, and T4 compared to T1 were 2.79, 8.27 and 8.75 respectively. These four histopathological parameters were then used to develop a predictive model for nodal metastasis. This model showed that probability of nodal metastasis is higher among tongue cancers with increasing POI, with increasing T, and with larger depths while other characteristics remained unchanged. The proposed model provides a way of using combinations of histopathological parameters to identify patients with higher risks of nodal metastasis for surgical management.

## Introduction

The incidence of oral squamous cell carcinoma (OSCC) is increasing globally and is a leading cause of death accounted for 8.8 million deaths in 2015 [1]. It is one of the most common cancers in Sri Lankan male population [2]. The survival rates have not improved significantly despite the advances in technology and treatment protocols. It is apparent that the high death rate relates to delayed diagnosis. The main attribute for above scenario is due to the fact that most oral cancers do not produce pain at early stage [2,3]. Tobacco use is the most important risk factor for cancer and is responsible for approximately 22% of cancer deaths [3].

The management of clinically negative neck nodes (N0) poses a significant challenge for surgeons, as there are no reliable parameters to predict occult metastasis. In order to identify patients who are likely to have nodal metastases, several parameters like tumour differentiation, perineural invasion, lymphovascular invasion, pattern of invasion (POI) and depth of invasion (DOI) have been previously studied [4,5]. According to our previous studies on OSCC, it has been shown that POI, tumour size (T) and stage are important parameters in predicting nodal metastasis [5,6]. Although DOI has been shown to be one of the important factors in predicting lymph node metastasis, it differs according to the sub site in the oral cavity. DOI is considered as an objective parameter and has been evaluated by several investigators for OSCC. Although most authors substantially agree that DOI is an important parameter for nodal metastasis and survival, the results vary in the literature and there is no cut-off point to prompt neck dissection [5]. One of the main etiological agents among patients from South Asian countries is betel quid, and many of them develop oral cancers if they have oral submucos fibrosis, hence the tumour invasive depth may differ due to fibrosis of the submucosa. Oral submucous fibrosis is a chronic, inflammatory disease characterized by progressive submucosal fibrosis of the mucosa and underlying connective tissues in the oral cavity and the oropharynx. People affected by this disease mostly live in south Asia, while migrants from these countries to the United States and Europe may also present with oral submucous fibrosis [5, 6].

Tumour thickness or the maximum depth of tumour infiltration is a well-established risk factor for many cancers mainly for gastrointestinal tract tumours [7]. However, there is no specific data available for oral cancer sub-sites. Some reports indicate that the rate of nodal metastasis differs according to the oral cavity sub-sites, and the thickness of invasion varies from 2-8mm between studies. Most of these studies discussed the relationship between DOI of the tongue, floor of the mouth and metastasis [7]. However, it is important to have data for the buccal mucosal cancers as it is the most common site affected in South and Southeast Asian patients.

The aetiological factors for OSCC show significant variation across different parts of the world. In the developed world it is mostly due to smoking, alcohol and human papilloma virus. However, in South and Southeast Asia OSCC is mostly related to betel chewing containing tobacco and arecanut [8]. Therefore, the most common site affected is buccal mucosa as they keep the betel quid in buccal pouch [5]. Further, most OSCCs are associated with oral submucous fibrosis, a potentially malignant disorder [8, 9]. Due to the difference in aetiology and extensive fibrosis, the relationship between tumour depth and metastasis may vary in patients with the history of betel chewing and those from the developed world. With this background we wanted to investigate the size of the tumour (T), POI and DOI in an attempt to propose a model for prediction of nodal metastasis, which may help surgeons to plan the management of clinically negative neck nodes in patients with OSCC. Having an accurate predictive model for oral cancer is important to identify patients that require extensive prophylactic surgical management of neck nodes. Such predictions would have an immediate clinical impact, especially to avoid unnecessary radical treatment of patients who are at a low risk of metastasis [5]. Further it is understood that cancers from two sites namely, buccal mucosa and tongue, have different rates of metastasis. Therefore, we planned to investigate the site, and incorporate it in predicting nodal metastasis [5]. The presence of cervical lymph node metastases is the most important prognostic factor for survival in patients with OSCC [5] hence prediction of metastasis will enable the surgeon to select the best mode of treatment to improve the prognosis. We therefore aimed to evaluate the association between DOI, POI and occurrence of metastasis of OSCC from buccal mucosa and tongue. We aim to (I) evaluate the associations between these clinical parameters and presence of nodal metastases, (II) develop a simple model for predicting the nodal metastasis (III) identify combinations of clinical parameters that can be used as a screening tool for nodal metastasis that can be used for Sri Lankan OSCC patients as well as in South Asian region.

## Results

Medical records and histological sections were available for 623 patients; of these nodal metastasis was observed for 189 (30%). Majority (76%) were males, 95% were aged 40 or more, 50% had cancer of the tongue, nearly half (47%) were in POI type IV with just one person in POI type I, and 11% were in T1 with near equal numbers in T2-T4 (Table 1). More than half (59%) of the depths were 5mm or more, corresponding percentage among 385 non-censored depth measurements was 53%. Chi-square test found the presence of nodal metastasis is associated with cancer site, T, POI, DOI, but not with age or sex (Table 1). Results were similar when this analysis was repeated with subsets of data for each site separately (results not included).

**Table 1:** Univariate relationships between clinical parameters and presence of nodal metastasis.

Clinical parameter	Cancer absent in nodes(N = 434)	cancer present in nodes(N = 189)	P-value (Chi-Square test)
	N (%)	N (%)	
**Age**			
40 or below	18 (64.3)	10 (35.7)	
41 or more	415 (70)	178 (30.0)	0.52
**Sex**			
female	106 (70.7)	44 (29.3)	
male	328 (69.3)	145 (30.7)	0.76
**Primary site of cancer**			
Buccal mucosa	239 (76.4)	74 (23.6)	
Tongue	195 (62.9)	115 (37.1)	<0.01
**Pattern of invasion (POI)**			
I	1 (100)	0 (0.0)	
II	92 (88.5)	12 (11.5)	
III	176 (78.2)	49 (21.8)	
IV	164 (56.2)	128 (43.8)	<0.01
**Size of the tumour (T)**			
T1	63 (92.6)	5 (7.4)	
T2	160 (81.6)	36 (18.4)	
T3	99 (60.4)	65 (39.6)	
T4	112 (58.0)	81 (42.0)	<0.01
**Depth of invasion (DOI)**			
1 to <2mm	38 (76.0)	12 (24.0)	
2 to <3mm	59 (79.7)	15 (20.3)	
3 to <4mm	64 (86.5)	10 (13.5)	
4 to <5mm	39 (75.0)	13 (25.0)	
5+mm	233 (63.3)	135 (36.7)	<0.01
**Depth (non-censored cases only, n = 385)**			
1 to <2mm	32 (74.4)	11 (25.6)	
2 to <3mm	49 (83.1)	10 (16.9)	
3 to <4mm	47 (87.0)	7 (13.0)	
4 to <5mm	21 (84.0)	4 (16.0)	
5+mm	137 (67.2)	67 (32.8)	0.009

Percentage (%) is the row percentage. POI type 1 group not used in Chi-Square test due to single person in it. Frequencies may not sum to total shown in column heading due to missing values.

After excluding 8 cases due to missing data and a single person in POI type I, 614 complete cases were included in the rest of the analyses. Univariate logistic regression found an odds ratio (OR) of 1.89 (95% CI: 1.33–2.69) for metastasis of tongue cancers relative to buccal mucosa (Table 2). OR for invasive patterns III and IV were 1.99 (95% CI: 1.01–3.95) and 5.83 (95% CI: 3.05–11.11) respectively relative to invasive pattern II. A similar dose-response relationship was also found with tumour size; OR for T2, T3, and T4 were 2.79 (95% CI: 1.05–7.44), 8.27 (95% CI: 3.15–21.70), 8.75 and (95% CI: 3.36–22.76) respectively relative to tumour size T1. Such a dose-response relationship was not observed with depth categories when either all cases were used or analyses were restricted only to non-censored cases. However, depth was found to be associated with presence of nodal metastasis (P<0.01). All these associations were statistically significant. However such associations were not found to be significant with regard to age or gender (Table 2).

**Table 2:** Strength of univariate associations between each characteristics and presence of nodal cancer (measured as univariate odds ratio) (N = 614).

Characteristic	OR	95% CI for OR		Pvalue
**Age**				
40 or below	Ref			
41 or more	0.76	0.34	1.68	0.50
**Sex**				
female	Ref			
male	1.06	0.71	1.59	0.78
**Primary site of cancer**				
Buccal mucosa	Ref			
Tongue	1.89	1.33	2.69	<0.01
**Pattern of invasion (POI)**				
II	Ref			
III	1.99	1.01	3.95	<0.01
IV	5.83	3.05	11.11	
**Size of the tumour (T)**				
T1	Ref			
T2	2.79	1.05	7.44	<0.01
T3	8.27	3.15	21.70	
T4	8.75	3.36	22.76	
**Depth of invasion(DOI)**				
1 to <2mm	Ref			
2 to <3mm	0.80	0.34	1.89	<0.01
3 to <4mm	0.48	0.19	1.22	
4 to <5mm	1.03	0.42	2.54	
5+mm	1.78	0.90	3.53	
**Depth (Non-censored cases only, n = 382)**				
1 to <2mm	Ref			
2 to <3mm	0.59	0.22	1.55	<0.01
3 to <4mm	0.42	0.15	1.20	
4 to <5mm	0.54	0.15	1.92	
5+mm	1.39	0.66	2.93	

OR = odds ratio, CI = confidence interval

**Fig 1:**
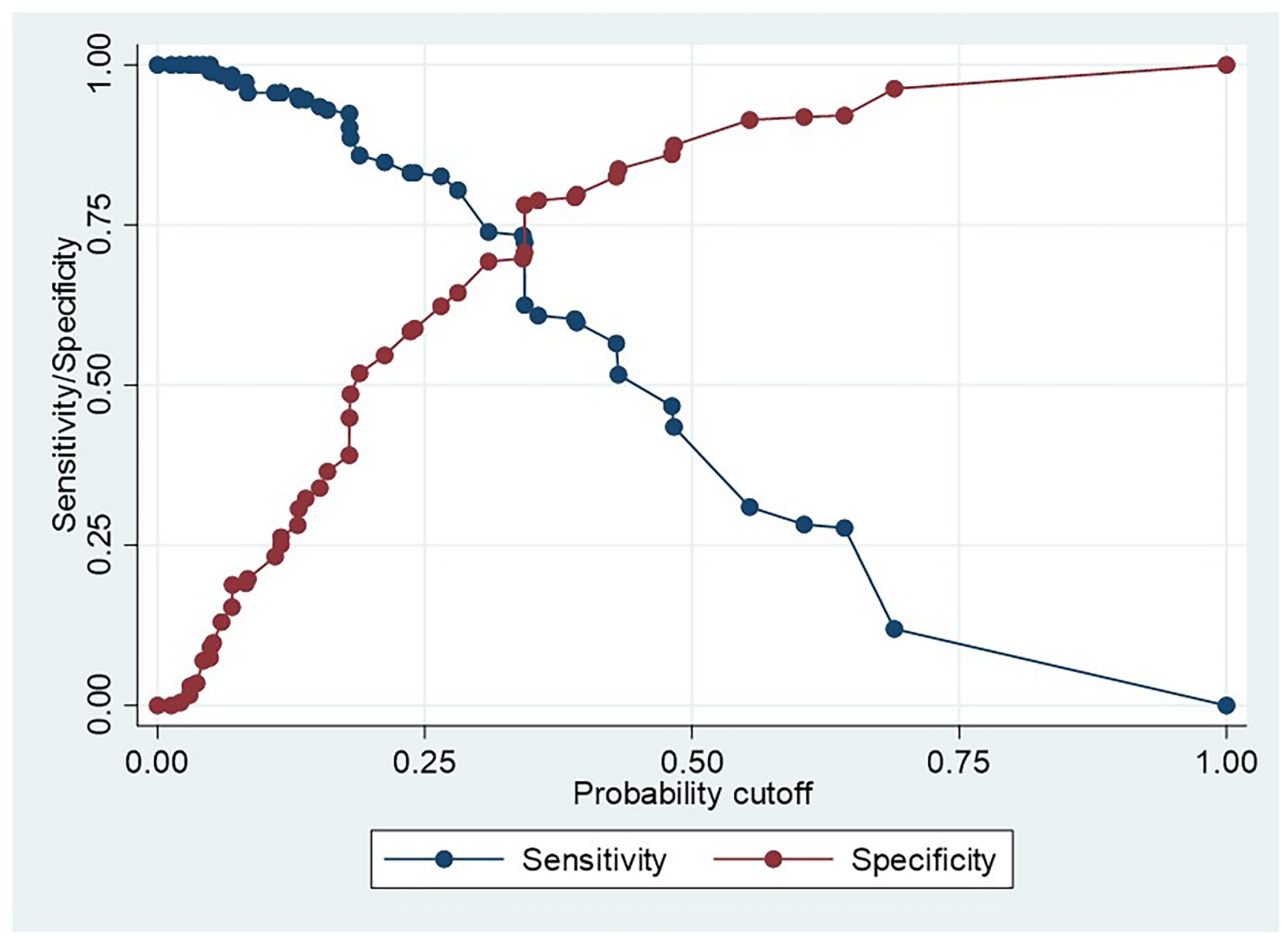
Sensitivity /specificity–probability cut off plot.

Based on univariate results, age and sex were not used in the multivariable model. The model fit of the multivariable predictive model was acceptable (Chi-square goodness of fit test *P-*value = 0.28, area under the ROC curve = 0.77). Probability of nodal metastasis predicted by the model for each of the 48 combinations of four clinical parameters are presented in Table 3. Tongue cancers were associated with a higher probability of nodal metastasis, so does the increasing POI, increasing T, and larger DOI (>4mm) when other characteristics remained unchanged. The table allows identifying combination of clinical characteristics with relatively higher (or lower) likelihood of having nodal metastasis than a probability level chosen by the practitioner. Fig 1 presents the sensitivity and specificity corresponds to such cut-off probabilities. For example, if one opt to select 0.25 probability of having metastasis as the cut-off for selecting those require neck dissections (ie, corresponds to pink or red cells in Table 3), figure reports corresponding sensitivity and specificity of that choice as about 83% and 63% respectively.

**Table 3:** Predicted probability of nodal metastasis when depth is dichotomised as <4mm and 4+mm.

Size of the Tumour (T)		Buccal mucosa			Tongue		
		POI 2	POI 3	POI 4	POI 2	POI 3	POI 4
T1	depth <4mm	0.01	0.02	0.05	0.03	0.05	0.11
	depth 4+ mm	0.02	0.03	0.07	0.04	0.07	0.15
T2	depth <4mm	0.04	0.06	0.13	0.08	0.13	0.27
	depth 4+ mm	0.05	0.08	0.18	0.12	0.18	0.34
T3	depth <4mm	0.12	0.18	0.34	0.24	0.34	0.55
	depth 4+ mm	0.16	0.24	0.43	0.31	0.43	0.64
T4	depth <4mm	0.14	0.21	0.39	0.28	0.39	0.60
	depth 4+ mm	0.19	0.28	0.48	0.36	0.48	0.69

POI- pattern of invasion, T- size of the tumour. Colour codes: white = probability is <0.10 (low risk), yellow = probability between 0.10 and 0.17 (minor risk), blue = probability between 0.18 and 0.24 (moderate risk), pink = probability between 0.25 and 0.34 (high risk), red = probability 0.35 or higher (severe risk).

## Discussion

Currently there are controversies in the management of clinically node-negative neck. Majority suggest elective neck dissections while some encourage close observation often with frequent ultrasound imaging [10]. Since late 1800s, radical neck dissection was performed to avoid cancer recurrences even though it caused significant post-operative complications such as shoulder dysfunction [11]. In 1980s more selective neck dissections were performed where the lymph nodes which drained the primary site of the tumour were removed. However sometimes it results in shoulder dysfunction despite of how conservative the management is. At present it is generally accepted that elective neck dissection is indicated for patients presenting with OSCC with a clinically N0 if the reported risk of occult metastases exceeds 15% - 20% [12]. Yet in most cases this may not be necessary [13]. In a study carried out by Shah et al 1990 revealed that even in the clinically node negative neck, nodal metastases were present in levels I-IV in up to 40% of neck dissections [14]. On the other hand, this describes that almost 60% of these patients have undergone unnecessary neck dissections.

To carry-out the most suitable management, PET/CT and CT and/or MRI imaging are being commonly used in developed countries to diagnose distant nodal metastasis in patients presenting with OSCCs [15]. Some authors suggest that PET/CT is a superior method when compared to CT/MRI [15], while others have found that it does not add much to a conventional imaging [16]. For example, neither PET/CT nor CT/MRI can identify micro metastases of 1-3mm in size [16]. Hence not only the use of such advance imaging modalities to detect metastases in clinically negative neck is controversial, it is not practical in most developing countries due to lack of modern facilities [5].

So far it is evident that the most significant determinant of overall survival in patients with OSCC is the cervical lymph node metastasis that is associated with 50% decrease in survival rate [15]. Due to the fact that detection of micro metastases and metastases of clinically negative neck is difficult, many cancer researchers were persuaded to develop predictive models to predict metastasis, recurrences and survival [17–20]. These predictive models were developed based on clinical, histopathological and molecular parameters related to cancers [17–20]. A group of researchers has developed and validated a prognostic model using gene profiles and clinicopathological co-variables [21]. Such models are difficult to be used in this part of the world since majority of the patients who present with OSCC are in low economic status and cannot afford expensive investigations such as gene profiling. Further in most diagnostic centers these advanced facilities are not available.

It is also worthwhile to mention that majority of OSCCs occur in South Asian population due to the habit of chewing betel quid with areca and due to the use of freeze dried areca products [8]. Arecoline in arecanut has been found to cause changes in the extracellular matrix of the oral submucosa, which can cause a potentially malignant disease like oral submucous fibrosis where the submucosa becomes fibrosed [8]. It is hypothesised that the dysplastic epithelium of OSF fails to invade the underlying connective tissue due to the fibrotic changes in the submucosa thus resulting in exophytic OSCCs [22]. Hence it can be argued whether the existing literature is reliable when treating OSCC patients in South Asia since the studies in the literature have used patients with different aetiological backgrounds. This may be true when assessing some important histopathological parameters such as depth of invasion and pattern of invasion in the background of oral submucous fibrosis [5, 8].

Our study specifically addressed this issue by using patients from a South Asian country to develop a model to predict the likelihood of nodal metastasis by using clinical and histopathologic parameters rather than depending on the existing literature.

Except DOI, all other data were extracted from patients’ records available in the Department of Oral Pathology, Faculty of Dental Sciences, University of Peradeniya, Sri Lanka. These reports were primarily designed to record data in order to facilitate histopathologic diagnosis of the disease and were not designed specifically for this study. Thus this study includes a secondary data analysis. Our data showed 70% of the patients did not have neck metastasis yet has undergone elective neck dissections (Table 1). This emphasizes the importance of constructing a method to predict or to identify patients who may have nodal metastasis and thereby to reduce unnecessary neck dissections in clinically negative necks.

Several studies suggest predictive models and scoring systems to predict nodal metastasis, recurrences and survival, using parameters such as gender, age, tumour site, tumour size, depth of invasion/ tumour thickness, pattern of invasion, perineural invasion, tumour grade (differentiation), lymphoid response, tumour budding, degree of keratinisation, nuclear pleomorphism, habits and gene profiling [23–27]. Therefore, we selected age, gender, primary site of the tumour, T, DOI, and POI as parameters to identify the association with nodal metastasis in order to propose a predictive model. In the present study it was evident that DOI (P<0.01), POI (P<0.01), primary site (P<0.01) and T (P<0.01) had significant associations with cervical nodal metastasis while age or gender did not show a significant association.

### Site of the tumour

When reviewing the literature, it was distinct that the tongue was considered as the most common site of oral cancer and exhibits high rates of metastasis outside South Asia while buccal cancers are much more predominant in South Asian countries [28–30]. Anatomically, the tongue is located on the floor of the oral cavity which is an area characterized by a rich supply of lymphatic vessels and neurovascular bundles. This is a reason to increase nodal metastasis in patients with tongue cancers. In contrast cancer in the buccal mucosa has a low rate of lymph-node metastasis [31]. The results of the current study showed that, although the number of cases are similar from two sites, higher proportion of nodal metastasis were observed in tongue cancers than in buccal mucosa cancers (37% vs 24%, P<0.01) (Table 1). The strength of this association was significant (OR = 1.89 CI 95% 1.33 to 2.69, P<0.01)) (Table 2).

### Size of the tumour

The greatest diameter of the tumour surface is used to indicate tumour size in the TNM classification. Controversies exist regarding this parameter where some authors suggest that increased tumour size has a link to cervical nodal metastasis [32–34], while some suggest that tumour size does not predict nodal metastasis [35]. Current study supports the former of these having a significant association between T and nodal metastasis. The likelihood of nodal metastasize is significantly higher with increasing tumour size showing a ‘dose-response relationship’; univariate odds ratios of metastasis are 2.79 (95% CI: 1.05 to 7.44), 8.27 (3.15 to 21.7), and 8.75 (3.36 to 22.76) for size T2, T3, and T4 respectively relative to size T1, (P<0.01) (Table 2).

### Pattern of invasion

The literature provides strong evidence for association of POI with local and distant nodal metastasis [17, 36–38]. Lim et al 2004 has reported that grade 3 or grade 4 pattern of invasion independently predicts late cervical metastasis in stage 1 and 2 OSCC (17). Another study has shown that POI was the most significant predictor for occult nodal metastasis [38]. Almangush et al 2014 has also reported similar findings [37]. Dissanayaka et al 2012 found a significant association between the pattern of invasion of the tumour and metastasis of the tumour to regional lymph nodes at presentation [39]. They have mentioned that tumours invading with POI 3 and 4 show a higher tendency to metastasize compared to tumours with POI 1 and 2 [39]. The results of the current study are consistent with above by pattern of invasion having a significant association with cervical nodal metastasis (Table 1), and also by showing a dose-response relationship between increasing type of POI and likelihood of nodal metastasis (Table 2).

### Depth of invasion

When reviewing the literature, it was noted that lack of standardized method of measuring tumour thickness/ depth of invasion has resulted in different cut off values to decide elective neck dissection for clinically negative neck. Some authors have used a technique developed by Breslow [40] where they have measured vertically, starting from the surface of the tumour or from the base of the ulcer to the deepest point of invasion [41–52]. While some have measured after constructing an imaginary line indicating the level of the adjacent intact oral mucosa [17, 53–57] or the basement membrane [58–61] up to the deepest point of invasion. Moore et al 1986 found that longer survival of patients with verrucous carcinoma was well associated with the thickness measured from the line of a basement membrane constructed through the tumour than with the complete thickness of the exophytic tumour [62]. Pentenero et al 2005 also suggested that it would be better to consider the actual mass beneath the basement membrane rather than measuring the entire thickness of the tumour [63]. Thus we decided to measure the depth of invasion starting from the intact basement membrane up to the point of deepest invasion.

Our descriptive (P<0.01, Table 1) and univariate (P<0.01, Table 2) analyses showed a significant association between DOI and nodal metastasis; however, in contrast to what were observed with POI and T, a dose-response relationship was not observed in this association. One possible reason for this could be the censored DOI measurements. In this study where the invasion in cancer tissues extended up to the inferior margin of the excisional biopsy specimen in H&E sections were only measured up to the inferior margin. Thus the DOI of about one third of the cases were under-measured (censored). This may have caused misclassification of DOI that suggests we may have underestimated the effect of DOI on the nodal metastasis because differential misclassification is known to bias the estimates negatively [64]. Therefore, the true effect of DOI likely be greater than what was reported by our results. Hence we carried out similar separate univariate analysis for uncensored measurements of DOI which revealed similar results (of odds ratios) (Table 2). Another possible reason for not observing a dose-response relationship could be the categorisations used for DOI (1mm categories). This prompted us to dichotomise the DOI for the predictive model using 4mm cut-off as those in less than 4mm DOI category were found to be associated with lower probability of nodal metastasis compared to others.

As mentioned before, different studies have suggested different cut-off values for DOI to predict metastasis for different sites in the oral cavity. Therefore, it is difficult to compare these cut off values due to the discrepancies of the methods used to measure the tumour thickness/ DOI. For example, certain studies which DOI has been measured starting from the adjacent intact mucosa have suggested 4mm [53, 56–58, 65] and 5mm [54, 55, 65] for tongue cancers and 2mm [66] for floor of the mouth cancers. On the other hand some other studies which have measured the tumour thickness starting from the surface of the tumour have suggested 4mm [49] for tongue cancers, 5mm [46] and 6mm [43] for cancers in the lip and 6mm [44] and 7mm [47] for overall oral cavity. In the present study the method which was used to measure DOI is similar to Giacomarra et al 1999 [61] and Ambrosch et al 1995 [58]. Their results revealed a DOI of 7mm [61] and 4mm [58] for cancers in upper aero digestive tract. Therefore, it is evident that DOI alone may not predict nodal metastasis with high accuracy. This demands a better method combining few other parameters to evaluate the clinically node negative neck and its management.

### Developing a predictive model

Results of the current study revealed strong associations between cervical nodal metastasis and the four parameters, site, T, POI and DOI. We hypothesised that the use of these parameters together may increase the accuracy of predicting nodal metastasis. Therefore, we used these four parameters to develop a multivariate logistic model which predicts possible presence of nodal metastasis more accurately. For reasons given above, and also to simplify the model, we dichotomised DOI as less than 4mm and equal or more than 4mm. Therefore, the predictive model included two sites, four categories of tumour size, two categories of DOI, and three grades of POI. Probability of nodal metastasis predicted by the model in Table 3 allows identifying combinations of clinical parameters which correspond to the likelihood of higher than any given probability threshold. Sensitivity-specificity plot in Fig 1 helps to assess the suitability of any such threshold. For example, say one intends to follow the 0.17 probability threshold as an approximation for the generally accepted probability of 15%-20% mentioned in first paragraph of discussion. Clinical parameters corresponds to that probability are identifiable from Table 3 as those in blue, pink, or red. Then using Fig 1 we can assess the sensitivity and specificity corresponding to that probability as 88% and 55% respectively. This means, using this threshold to identify patients for neck dissections may not include 12% of the patients who have nodal metastasis, while carrying out unnecessary dissections in 45% of those without nodal metastasis.

In order to predict the presence of nodal metastasis and to avoid under treatment the sensitivity of the cut off value must be high. At the same time, it should have a considerable specificity to reduce over-treatment (unnecessary neck dissections). Hence this value requires a compromise between sensitivity and specificity [67]. We do not intend to make recommendation on the suitability or otherwise of any probability threshold as it is not within the scope of this study.

The final outcome of the model revealed that T4 tumours in the buccal mucosa that have POI type 3 and has a depth of invasion > 4 mm are more likely to metastasize. It also showed that T3 and T4 tumours in the buccal mucosa with POI type 4 tend to metastasize regardless of its depth of invasion.

The model showed that there is a high chance of nodal metastasis when there is a tumour on the tongue that has a POI of type 4 regardless of depth of invasion. T3 tumours in the tongue tend to metastasize when the POI is 2 and DOI is more than 4mm. T3 tumours also has a high chance of metastasis when the POI is type 3 or 4 regardless of its DOI. The model also revealed that T4 tongue tumours have a high chance of cervical nodal metastasis regardless of its DOI and POI.

### Strengths, limitations and conclusion

This study proposes a simple predictive model for the risk of nodal metastasis in clinically negative necks. The major drawback of this study was the difficulty in measuring exact DOI in significant number of cases. However, the study has strength that, it is based on a large sample, proposed model being simple, and based on parameters empirically supported as well as established in literature, easy to use in routine clinical practice, and cost effective. Although findings of this retrospective investigation needs required to be validated in a prospective study, it provides a useful model that is practicable to use in developing countries of South and Southeast Asia where OSCC aetiology is similar and advanced facilities are not available.

## Materials and methods

**Fig 2:**
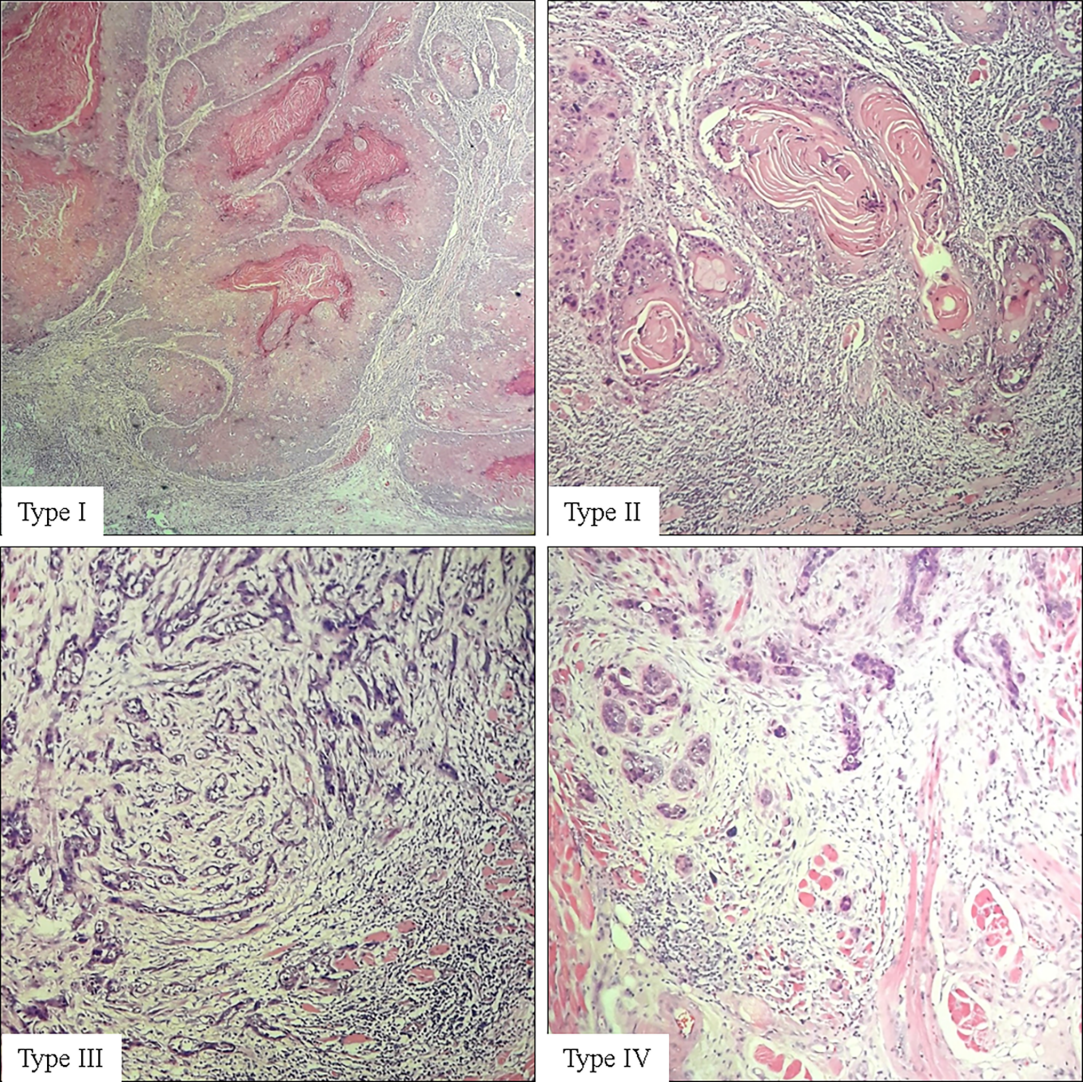
Different invasive patterns of oral squamous cell carcinoma.

**Fig 3:**
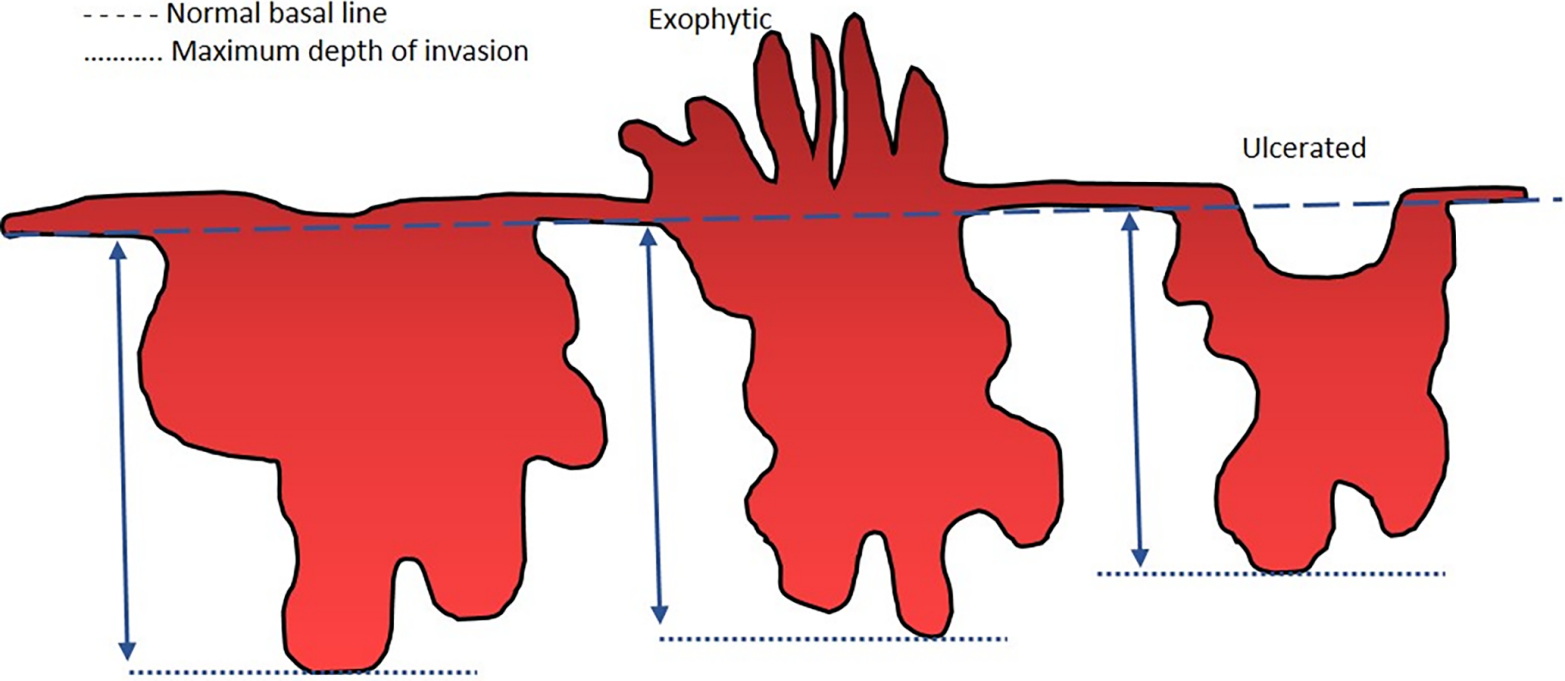
Representative schema of tumour depth and measurement of depth of invasion.

All patients who underwent surgery for OSCC of the tongue or buccal mucosa with neck dissection between 1998 and 2015 were retrospectively studied. The cases were selected from archives of the Department of Oral Pathology, Faculty of Dental Sciences, University of Peradeniya, the only Head and neck Pathology centre which receives most biopsies from almost all Oral and Maxillofacial units in Sri Lanka. Therefore this can be considered as a representative sample of the entire country. Demographic data including age, gender and clinical information such as size of the tumour (T), primary site; tongue or buccal mucosa, and pathological factors (POI, lymph node metastasis) were recorded. Arrangement of tumour cells at the advancing front was graded as large cohesive tumour islands (POI- I), small islands (POI- II), thin strands (POI- III) and individual tumour cells (POI- IV) [68] (Fig 2). Eight cases (<15%) were excluded due to missing data on one or more of above parameters. Patients with recurrent tumours or extensive tumours involving multiple intraoral sites were not included. DOI was obtained from histological sections following the standard method of measuring the distance from the deepest point of infiltration to the basal layer of overlying epithelium using an optical micrometer with a graticule [58, 61]. The depth (0.25mm accuracy) was taken from an imaginary line at the basement membrane of the healthy oral mucosa to the deepest point of tumour invasion (Fig 3A and 3B). Multiple sections of the tumour were measured to identify the region with maximum thickness. All data were anonymised before analysis and informed consent has been taken from all patients to use their information/ material for research. Ethical clearance for the study was obtained from the Faculty Research and Ethical Review Committee (ERC/FDS/UoP/I/2016/05).

### Data analysis

Patient’s histological sections had been prepared primarily for managing with curative intent; therefore full width of the tumour had not been taken to a single section in about one third of the sections. Since the DOI was measured from the basement membrane of the healthy oral mucosa, section containing this may not contained the full depth of the tumour in these cases which prevented from accurate measurement of the depth of the tumour. We recorded the DOI of these sections and used in the analysis as the lower end for their actual tumour depth knowing that these are under-measurements of the actual thickness of these cases. Those measurements are hereafter referred to as censored depths.

For the purpose of analysis, age was dichotomised as 40 years or below vs 41 or more years. Depth was initially categorised into five groups; four 1mm groups and one group containing cases with 5mm or more depths (Table 1). Association between each variable and presence of nodal metastasis was assessed using chi-square test. Univariate logistic regression was used to estimate the strength of these associations in terms of odds ratios (and 95% confidence intervals, CIs). Based on univariate results for developing a predictive model, we dichotomised depth as less than 4mm, and 4mm or more. Clinical parameters identified in univariate assessments were then used in multivariable logistic regression to estimate the probability of having nodal metastasis for each combinations of parameters. These probabilities were then used to rank combinations of parameters on the basis of associated risk of having nodal metastasis. The number of cases with missing data was small (<1.5%), thus all statistical modelling was done as complete case analyses using Stata 15.1® software [69].

## Supporting information

S1 Dataset Raw dataset. (XLS)
